# Supplementary material for: Modeling the circadian regulation of the immune system: Sexually dimorphic effects of shift work
Source: PLoS Comput Biol. 2021 Mar 31;17(3):e1008514. doi: 10.1371/journal.pcbi.1008514 (PMC8041207; doi:10.1371/journal.pcbi.1008514)
Supplement: S1 File — (Table A) List of variables, (Table B) list of the differential equations defining the mathematical model; relates to Fig 1. (Tables C-J) Nominal parameter set of the mathematical model. (Tables K-M) Parameter values in CJL models. Supplemental experimental procedure: Sobol’ sensitivity analysis. (PDF) [file pcbi.1008514.s001.pdf]

Supporting Information

Modeling the circadian regulation of the immune system: sexually dimorphic effects of shiftwork

Stéphanie M.C. Abo[1*], Anita T. Layton[1,2]

1 Department of Applied Mathematics, University of Waterloo, Waterloo, Ontario, Canada
2 Department of Biology and Schools of Computer Science and Pharmacology, University of Waterloo, Waterloo, Ontario, Canada

Inventory of supporting information

Supplemental tables

Table A List of variables of the mathematical model. Relates to Fig 1.

Table B Differential equations defining the mathematical model.

Table C-J List of the kinetic constants obtained by parameter identification.

Table K List of kinetic constants modified in CJL models.

Table L Percentage change in mean gene expression level due t CJL.

Table M Parameters studied for the Sobol' sensitivity analysis.

The Sobol' sensitivity analysis

Fig A Sobol' indices for parameters modified by CJL.

Fig B Time course of total-order Sobol' indices for vmax_cry, kass_pc and kass_cb.

Fig C Computed simulation results for D, IL-6, TNF-α and IL-10.

Fig D Sobol' indices for the coupling parameters.

Fig E Time course of total-order Sobol' indices for the coupling parameters.

1 Supplemental Tables

Table A. List of variables. We adopt the notation where names with a mix of upper and lower case letters (e.g., Per) denote mRNAs, and names in all caps (e.g., PER) denote proteins.

Variable name	Description
Per	Concentration of *Per* mRNA
Cry	Concentration of *Cry* mRNA
Rev-Erb	Concentration of *Rev-Erb* mRNA
Ror	Concentration of *Ror* mRNA
Bmal1	Concentration of *Bmal1* mRNA
PER	Concentration of *PER* protein
CRY	Concentration of *CRY* protein
REV-ERB	Concentration of *REV-ERB* protein
ROR	Concentration of *ROR* protein
BMAL1	Concentration of *BMAL1* protein
PER-CRY	Concentration of *PER-CRY* protein
CLOCK-BMAL1	Concentration of *CLOCK-BMAL1* protein
P	Concentration of LPS
N	Total number of activated phagocytic cells
D	Tissue damage marker
TNF	Concentration of *tumor necrosis factor-α*
IL6	Concentration of *interleukin-6*
IL10	Concentration of *interleukin-10*
Y_{IL10}	Tissue damage driven non-accessible *interleukin-10* promoter
CA	Concentration of slow-acting anti-inflammatory mediators

Table B. Differential equations defining the mathematical model.

Circadian genes and proteins (Eqs.1-12)

$$\frac{\mathrm{d}Per(t)}{\mathrm{d}t} = -dm\_per \cdot Per(t) + \frac{vmax\_per \cdot \left(1 + fold\_per \cdot \left(\frac{CLOCK\text{-}BMAL1(t)}{ka\_per\_cb}\right)^{hill\_per\_cb}\right)}{1 + \left(\frac{CLOCK\text{-}BMAL1(t)}{ka\_per\_cb}\right)^{hill\_per\_cb} \cdot \left(1 + \left(\frac{PER\text{-}CRY(t)}{ki\_per\_pc}\right)^{hill\_per\_pc}\right)}, \tag{1}$$

$$\frac{\mathrm{d}Cry(t)}{\mathrm{d}t} = -dm\_cry \cdot Cry(t) + \frac{1}{\left(1 + \left(\frac{REV\text{-}ERB(t)}{ki\_cry\_rev}\right)^{hill\_cry\_rev}\right)} \cdot \frac{vmax\_cry \cdot \left(1 + fold\_cry \cdot \left(\frac{CLOCK\text{-}BMAL1(t)}{ka\_cry\_cb}\right)^{hill\_cry\_cb}\right)}{1 + \left(\frac{CLOCK\text{-}BMAL1(t)}{ka\_cry\_cb}\right)^{hill\_cry\_cb} \cdot \left(1 + \left(\frac{PER\text{-}CRY(t)}{ki\_cry\_pc}\right)^{hill\_cry\_pc}\right)}, \tag{2}$$

$$\frac{\mathrm{d}Rev\text{-}Erb(t)}{\mathrm{d}t} = -dm\_rev \cdot Rev\text{-}Erb(t) + \frac{vmax\_rev \cdot \left(1 + fold\_rev \cdot \left(\frac{CLOCK\text{-}BMAL1(t)}{ka\_rev\_cb}\right)^{hill\_rev\_cb}\right)}{1 + \left(\frac{CLOCK\text{-}BMAL1(t)}{ka\_rev\_cb}\right)^{hill\_rev\_cb} \cdot \left(1 + \left(\frac{PER\text{-}CRY(t)}{ki\_rev\_pc}\right)^{hill\_rev\_pc}\right)}, \tag{3}$$

$$\frac{\mathrm{d}Ror(t)}{\mathrm{d}t} = -dm\_ror \cdot Ror(t) + \frac{vmax\_ror \cdot \left(1 + fold\_ror \cdot \left(\frac{CLOCK\text{-}BMAL1(t)}{ka\_ror\_cb}\right)^{hill\_ror\_cb}\right)}{1 + \left(\frac{CLOCK\text{-}BMAL1(t)}{ka\_ror\_cb}\right)^{hill\_ror\_cb} \cdot \left(1 + \left(\frac{PER\text{-}CRY(t)}{ki\_ror\_pc}\right)^{hill\_ror\_pc}\right)}, \tag{4}$$

$$\frac{\mathrm{d}Bmal1(t)}{\mathrm{d}t} = -dm\_bmal \cdot Bmal1(t) + \frac{x_P}{x_P + F_P(t)} \cdot \frac{vmax\_bmal \cdot \left(1 + fold\_bmal \cdot \left(\frac{ROR(t)}{ka\_bmal\_ror}\right)^{hill\_bmal\_ror}\right)}{1 + \left(\frac{REV\text{-}ERB(t)}{ka\_bmal\_rev}\right)^{hill\_bmal\_rev} + \left(\frac{ROR(t)}{ka\_bmal\_ror}\right)^{hill\_bmal\_ror}}, \tag{5}$$

$$\frac{\mathrm{d}PER(t)}{\mathrm{d}t} = -\,dp\_per \cdot PER(t) + kp\_per \cdot Per(t) - \left[kass\_pc \cdot PER(t) \cdot CRY(t) - kdiss\_pc \cdot PER\text{-}CRY(t)\right], \tag{6}$$

$$\frac{\mathrm{d}CRY(t)}{\mathrm{d}t} = -\,dp\_cry \cdot CRY(t) + kp\_cry \cdot Cry(t) - \left[kass\_pc \cdot PER(t) \cdot CRY(t) - kdiss\_pc \cdot PER\text{-}CRY(t)\right], \tag{7}$$

$$\frac{\mathrm{d}REV\text{-}ERB(t)}{\mathrm{d}t} = -dp\_rev \cdot REV\text{-}ERB(t) + kp\_rev \cdot Rev\text{-}Erb(t), \tag{8}$$

$$\frac{\mathrm{d}ROR(t)}{\mathrm{d}t} = -dp\_ror \cdot ROR(t) + kp\_ror \cdot Ror(t), \tag{9}$$

$$\frac{\mathrm{d}BMAL1(t)}{\mathrm{d}t} = -\,dp\_bmal \cdot BMAL1(t) + kp\_bmal \cdot Bmal1(t) - kass\_cb \cdot BMAL1(t) + kdiss\_cb \cdot CLOCK\text{-}BMAL1(t), \tag{10}$$

$$\frac{\mathrm{d}PER\text{-}CRY(t)}{\mathrm{d}t} = kass\_pc \cdot PER(t) \cdot CRY(t) - kdiss\_pc \cdot PER\text{-}CRY(t) - d\_pc \cdot PER\text{-}CRY(t), \tag{11}$$

$$\frac{\mathrm{d}CLOCK\text{-}BMAL1(t)}{\mathrm{d}t} = \left[kass\_cb \cdot BMAL1(t) - kdiss\_cb \cdot CLOCK\text{-}BMAL1(t)\right] - d\_cb \cdot CLOCK\text{-}BMAL1(t), \tag{12}$$

Immune system agents (Eqs.13-20)

Endotoxin concentration

$$\frac{\mathrm{d}P(t)}{\mathrm{d}t} = -d_p \cdot P(t), \tag{13}$$

The endotoxin insult injected intraperitoneally in the rats is a bolus administration, which initiates the inflammatory cascade. The initial conditions for P are either 3, 6, or 12 mg/kg depending on the endotoxin dose level.

Total number of activated phagocytic cells

$$\frac{\mathrm{d}N(t)}{\mathrm{d}t} = k_N \cdot \Big(\frac{R(t)}{x_N + R(t)}\Big) - d_N \cdot N(t), \tag{14}$$

$$\begin{aligned}
R(t) &= \big[k_{NP} \cdot P(t) + k_{ND} \cdot D(t)\big] \cdot fDN_{NCA}(t) \cdot fDN_{NIL10}(t) \\
&\quad \cdot (1 + k_{NTNF} \cdot fUP_{NTNF}(t)) \cdot (1 + k_{NIL6} \cdot fUP_{NIL6}(t)) \\
fUP_{NTNF}(t) &= \frac{TNF(t)}{x_{NTNF} + TNF(t)} \\
fUP_{NIL6}(t) &= \frac{IL6(t)}{x_{NIL6} + IL6(t)} \\
fDN_{NCA}(t) &= \frac{xNCA}{x_{NCA} + CA(t)} \\
fDN_{NIL10}(t) &= \frac{xNIL10}{x_{NIL10} + IL10(t)}
\end{aligned}$$

The initial condition is $N(0) = 0$.

Tissue damage marker

$$\frac{\mathrm{d}D(t)}{\mathrm{d}t} = k_D \cdot \frac{IL6(t)^4}{x_D^4 + IL6(t)^4} + k_P \frac{P(t)}{x_P + P(t)} - d_D \cdot D(t), \tag{15}$$

The initial condition is $D(0) = 0$.

Concentration of interleukin-6

$$\begin{aligned}
\frac{\mathrm{d}IL\text{-}6(t)}{\mathrm{d}t} &= k_{IL6} \cdot \frac{N(t)^4}{x_{IL6}^4 + N(t)^4} \cdot fDN_{IL6IL10}(t) \cdot fDN_{IL6CA}(t) \cdot fDN_{IL6REV}(t) \\
&\quad \cdot \big[1 + k_{IL6TNF} \cdot fUP_{IL6TNF}(t) + k_{IL6IL6} \cdot fUP_{IL6IL6}(t)\big] - d_{IL6} \cdot IL6(t),
\end{aligned} \tag{16}$$

$$\begin{aligned}
fUP_{IL6TNF}(t) &= \frac{TNF(t)}{x_{IL6TNF} + TNF(t)} \\
fUP_{IL6IL6}(t) &= \frac{IL6(t)}{x_{IL6IL6} + IL6(t)} \\
fDN_{IL6IL10}(t) &= \frac{x_{IL6IL10}}{x_{IL6IL10} + IL10(t)} \\
fDN_{IL6CA}(t) &= \frac{x_{IL6CA}}{x_{IL6CA} + CA(t)} \\
fDN_{IL6REV}(t) &= \frac{x_{IL6REV}}{x_{IL6REV} + REV(t)}
\end{aligned}$$

The initial condition is *IL-6(0)=0*.

Concentration of tumor necrosis factor α

$$\frac{\mathrm{d}TNF\text{-}\alpha(t)}{\mathrm{d}t} = k_{TNF} \cdot N(t)^{1.5} \cdot \left[1 + k_{TNFTNF} \cdot fUP_{TNFTNF}(t)\right] \cdot fDN_{TNFCA}(t) \cdot fDN_{TNFIL10}(t) \cdot fDN_{TNFIL6}(t) \cdot fDN_{TNFCRY}(t) \cdot fDN_{TNFROR}(t) - d_{TNF} \cdot TNF(t), \tag{17}$$

$$fUP_{TNFTNF}(t) = \frac{TNF(t)}{x_{TNFTNF} + TNF(t)}$$
$$fDN_{TNFCA}(t) = \frac{x_{TNFCA}^6}{x_{TNFCA}^6 + CA(t)^6}$$
$$fDN_{TNFIL10}(t) = \frac{x_{TNFIL10}}{x_{TNFIL10} + IL10(t)}$$
$$fDN_{TNFIL6}(t) = \frac{x_{TNFIL6}}{x_{TNFIL6} + IL6(t)}$$
$$fDN_{TNFCRY}(t) = \frac{x_{TNFCRY}}{x_{TNFCRY} + CRY(t)}$$
$$fDN_{TNFROR}(t) = \frac{x_{TNFROR}}{x_{TNFROR} + ROR(t)}$$

The initial condition is $TNF\text{-}\alpha(0) = 0$.

Concentration of interleukin-10

$$\frac{\mathrm{d}IL10(t)}{\mathrm{d}t} = k_{IL10} \cdot \frac{N(t)^3}{x_{IL10}^3 + N(t)^3} \cdot \left[1 + k_{IL10IL6} \cdot fUP_{IL10IL6}(t) + k_{IL10TNF} \cdot fUP_{IL10TNF}(t)\right] \cdot fDN_{IL6REV}(t) - d_{IL10} \cdot fDN_{IL10d}(t) + Y_{IL10}(t) + s_{IL10}, \tag{18}$$

$$fUP_{IL10IL6}(t) = \frac{IL6(t)^4}{x_{IL10IL6}^4 + IL6(t)^4}$$
$$fUP_{IL10TNF}(t) = \frac{TNF(t)}{x_{IL10TNF} + TNF(t)}$$
$$fDN_{IL10d}(t) = \frac{x_{IL10d}}{x_{IL10d} + IL10(t)}$$
$$fDN_{IL10REV}(t) = \frac{x_{IL10REV}}{x_{IL10REV} + REV(t)}$$

The production of *IL-10* in the basal state is represented by the constant $sIL10$. With $N(0) = 0$, the initial condition is $IL\text{-}10(0) = \frac{s_{IL10} \cdot x_{IL10d}}{d_{IL10} \cdot x_{IL10d} - s_{IL10}}$.

Tissue damage driven non-accessible interleukin-10 promoter

$$\frac{\mathrm{d}Y_{IL10}(t)}{\mathrm{d}t} = k_{IL102} \cdot \frac{D(t)^4}{x_{IL102}^4 + D(t)^4} - d_{IL102} \cdot Y_{IL10}(t), \tag{19}$$

The initial condition is $Y_{IL10}(0) = 0$.

Anti-inflammatory moderator

$$\frac{\mathrm{d}CA(t)}{\mathrm{d}t} = k_{CA} \cdot N(t) - d_{CA} \cdot CA(t) + s_{CA}, \tag{20}$$

At basal conditions, the system is assumed to be slightly anti-inflammatory. This was achieved by introducing a constant, sCA, into the ODE. Hence, with $N(0) = 0$, we obtain $CA(0) = \frac{sCA}{dCA}$.

LPS filter

$$F_P(t) = P_0 - \frac{P_0}{24}t. \tag{21}$$

where $P_0 = P(t = 0)$. $F_P(0)$ is either 3, 6, or 12 mg/kg depending on the endotoxin dose level.

Table C. mRNA and protein degradation rate constants (in h^{-1})

Parameter	Value	Description
dm_per	0.10576	*Per* mRNA degradation rate constant
dm_cry	0.50633	*Cry* mRNA degradation rate constant
dm_rev	0.47914	*Rev-Erb* mRNA degradation rate constant
dm_ror	0.26786	*Ror* mRNA degradation rate constant
dm_bmal	4.6995	*Bmal1* mRNA degradation rate constant
dp_per	0.14989	*PER* protein degradation rate constant
dp_cry	1.9105	*CRY* protein degradation rate constant
dp_rev	0.28899	*REV-ERB* protein degradation rate constant
dp_ror	0.063637	*ROR* protein degradation rate constant
dp_bmal	0.22534	*BMAL1* protein degradation rate constant
d_pc	0.22571	*PER-CRY* protein complex degradation rate constant
d_cb	0.1709	*CLOCK-BMAL1* protein complex degradation rate constant

Table D. Maximal transcription rates (in $nmol \cdot l^{-1} \cdot h^{-1}$)).

Parameter	Value	Description
vmax_per	0.83525	*Per* mRNA maximal transcription rate
vmax_cry	1.0418	*Cry* mRNA maximal transcription rate
vmax_rev	0.065746	*Rev-Erb* mRNA maximal transcription rate
vmax_ror	7.2287	*Ror* mRNA maximal transcription rate
vmax_bmal	0.29055	*Bmal1* mRNA maximal transcription rate

Table E. Activation ratios (dimensionless)

Parameter	Value	Description
fold_per	0.12156	Activation ratio of *Per* by *CLOCK-BMAL1*
fold_cry	13.828	Activation ratio of *Cry* by *CLOCK-BMAL1*
fold_rev	130.78	Activation ratio of *Rev-Erb* by *CLOCK-BMAL1*
fold_ror	0.078569	Activation ratio of *Ror* by *CLOCK-BMAL1*
fold_bmal	43.306	Activation ratio of *Bmal1* by *ROR*

Table F. Regulation thresholds (in *nmol/l*)

Parameter	Value	Description
Ka_per_cb	3.3679	Regulation threshold of *Per* by *CLOCK-BMAL1*
Ki_per_pc	0.14178	Regulation threshold of *Per* by *PER-CRY*
Ka_cry_cb	1.5508	Regulation threshold of *Cry* by *CLOCK-BMAL1*
Ki_cry_pc	0.0027556	Regulation threshold of *Cry* by *PER-CRY*
Ki_cry_rev	0.64066	Regulation threshold of *Cry* by *REV-ERB*
Ka_rev_cb	0.18454	Regulation threshold of *Rev-Erb* by *CLOCK-BMAL1*
Ki_rev_pc	550.46	Regulation threshold of *Rev-Erb* by *PER-CRY*
Ka_ror_cb	0.56517	Regulation threshold of *Ror* by *CLOCK-BMAL1*
Ki_ror_pc	0.072928	Regulation threshold of *Ror* by *PER-CRY*
Ka_bmal_ror	0.076498	Regulation threshold of *Bmal1* by *ROR*
Ki_bmal_rev	0.0002375	Regulation threshold of *Bmal1* by *REV-ERB*

Table G. Hill coefficients (dimensionless)

Parameter	Value	Description
hill_per_cb	17.025	Hill coefficient regulation of *Per* by *CLOCK-BMAL1*
hill_per_pc	22.829	Hill coefficient regulation of *Per* by *PER-CRY*
hill_cry_cb	7.4632	Hill coefficient regulation of *Cry* by *CLOCK-BMAL1*
hill_cry_pc	2.583	Hill coefficient regulation of *Cry* by *PER-CRY*
hill_cry_rev	58.733	Hill coefficient regulation of *Cry* by *REV-ERB*
hill_rev_cb	9.3373	Hill coefficient regulation of *Rev-Erb* by *CLOCK-BMAL1*
hill_rev_pc	0.95847	Hill coefficient regulation of *Rev-Erb* by *PER-CRY*
hill_ror_cb	6.0371	Hill coefficient regulation of *Ror* by *CLOCK-BMAL1*
hill_ror_pc	3.2993	Hill coefficient regulation of *Ror* by *PER-CRY*
hill_bmal_ror	2.8187	Hill coefficient regulation of *Bmal1* by *ROR*
hill_bmal_rev	1.5678	Hill coefficient regulation of *Bmal1* by *REV-ERB*

Table H. Translation rates (in molecules per hour per mRNA)

Parameter	Value	Description
kp_per	0.77741	*Per* translation rate
kp_cry	0.9308	*Cry* translation rate
kp_rev	0.0004355	*Rev-Erb* translation rate
kp_ror	0.010866	*Ror* translation rate
kp_bmal	0.97306	*Bmal1* translation rate

Table I. Complexation kinetic rates

Parameter	Value	Unit	Description
kass_cb	0.0057803	h^{-1}	*CLOCK-BMAL* association rate
kass_pc	0.15187	$nmol{\cdot}l^{-1} \cdot h^{-1}$	*PER-CRY* association rate
kdiss_cb	0.00022191	h^{-1}	*CLOCK-BMAL* disassociation rate
kdiss_pc	0.23509	h^{-1}	*PER-CRY* disassociation rate

Table J. Parameters of the inflammation model.

Parameter	Value	Unit
**Endotoxin**		
d_p	3	h^{-1}
**Phagocytes**		
k_N	5.239009955e+07	h^{-1}
x_N	11.5345	N-unit
d_N	0.195335	h^{-1}
k_{NP}	46.8879	N-unit × kg/mg
k_{ND}	0.01297224	N-unit/D-unit
x_{NTNF}	1530.0904	pg/ml
x_{NIL6}	52121.3480	pg/ml
x_{NCA}	0.0819918	pg/ml
x_{NIL10}	138.3830	pg/ml
k_{NTNF}	15.7694	
k_{NIL6}	2.916366	
**Damage**		
k_D	0.747386	D-unit/h
d_D	0.434761	h^{-1}
x_D	3572.1137	pg/ml
k_P	1.385458	D-unit/h
x_P	0.5746	mg/kg
**Slow-acting cytokines**		
k_{CA}	1.381866e-09	pg/(ml × h × N-unit)
d_{CA}	3.1777e-2	h^{-1}
s_{CA}	0.004	pg/(ml × h)
**IL-6**		
k_{IL6TNF}	23.15473	
x_{IL6TNF}	1072.9657	pg/ml
k_{IL6}	4.2094572e+07	pg/(ml × h)
d_{IL6}	0.410396	h^{-1}
x_{IL6}	2.012412e+08	N-unit
$x_{IL6IL10}$	1.32377	pg/ml
k_{IL6IL6}	101.1321	
x_{IL6IL6}	14308.8692	pg/ml
x_{IL6CA}	1.104116	pg/ml
**TNF-α**		
k_{TNF}	9.326669e-08	pg/(ml × N-unit$^{1.5}$)
d_{TNF}	1.99835	h^{-1}
$x_{TNFIL10}$	6177.1302	pg/ml
x_{TNFCA}	0.223434	pg/ml
k_{TNFTNF}	0.198227	
x_{TNFTNF}	8520.5658	pg/ml
x_{TNFIL6}	40998.1848	pg/ml
**IL-10**		
$k_{IL10TNF}$	0.212173	
$x_{IL10TNF}$	8905.7477	pg/ml
$k_{IL10IL6}$	3.27267	
$x_{IL10IL6}$	22345.6179	pg/ml
k_{IL10}	1.9301e+05	pg/(ml × h)
d_{IL10}	95.465	h^{-1}
x_{IL10}	5.938865e+07	N-unit
s_{IL10}	1187.2	pg/(ml × h)

x_{IL10d}	713.8094	pg/ml
$\mathbf{Y}_{IL10}$		
k_{IL102}	3.804797e+06	Y_{IL10}-unit/h
d_{IL102}	0.0224238	h^{-1}
x_{IL102}	8.470849	D-unit
**coupling parameters**		
x_{IL6REV}	0.009	*nmol/l*
$x_{IL10REV}$	0.004	*nmol/l*
x_{TNFROR}	0.4534	*nmol/l*
x_{TNFCRY}	0.4315	*nmol/l*

Table K. List of parameters modified in CJL models.

Parameter	Control	CJL male	CJL female
vmax_per	0.83525	2.1717	2.9568
vmax_cry	1.0418	unchanged	1.6669
vmax_rev	0.065746	0.1249	0.0164
vmax_bmal	0.29055	unchanged	0.1511
kdiss_pc	0.23509	0.6112	1.3315
kdiss_cb	2.2191e-4	unchanged	1.1539e-4
kass_pc	0.15187	0.0584	0.0268
kass_cb	0.0057803	unchanged	0.0111
Ki_bmal_rev	2.375e-4	4.5125e-4	5.9375e-5
Ki_cry_rev	0.64066	1.2173	0.1602

Table L. Percentage change in mean gene expression level due to CJL

Gene	CJL male	Experimental data [1]	CJL female	Experimental data [1]
Bmal1	+3%	Not significant	−44%	−43% ± 38%
Per2	+236%	+230% ± 201%	+510%	+497% ± 234%
Cry2	+2%	Not significant	+69%	+69% ± 18%
Rev-Erbα	+103%	+98% ± 96.5%	−72%	−70% ± 23%
Ror	+8%	Not recorded	+19%	Not recorded

2 The Sobol' sensitivity analysis

The method of Sobol' [2] is a global and model independent sensitivity analysis method that is based on variance decomposition. Variance-based measures of sensitivity are attractive because they measure sensitivity across the whole input space, they can handle non-linear and non-monotonic functions and models. They can also measure the effect of interactions in such systems.

A first order index, S_i, is a measure for the variance contribution of the individual parameter to the total model variance, whereas a total order index, S_{Ti} is the result of the main effect of a given parameter and all its interactions with the other parameters. Note that for non-additive models as ours, interactions exist: S_{Ti} is greater than S_i and the sum of all S_i is less than 1. On the other hand, the sum of all S_{Ti} is greater than 1. By analyzing the difference between S_{Ti} and S_i, one can determine the impact of the interactions between a parameter of interest and the other parameters [3]. In this work, we will not discuss how to implement the Sobol' method, we refer to [2, 3] and the references therein.

2.1 The parameters considered for the sensitivity analysis

Based on our CJL male and CJL female models, 10 parameters are selected for the initial Sobol' sensitivity analysis. The set includes all parameters which took new values after fitting the CJL models. Lower and upper bounds to the parameter input space are obtained by halving and doubling nominal values, respectively (see Table M). Our outputs of interest are IL-6, TNF-α, IL-10 and D because they convey the most information about the state inflammation, and are also directly affected by the circadian clock model. For each output, we report first and total order effects. We performed 5000×(2+ number of input parameters) simulations to derive the Sobol' indices.

Table M. Parameters studied for the Sobol' sensitivity analysis

Parameter	nominal value	lower bound	upper bound
v_{max}^{per}	0.83525	0.4176	1.6705
v_{max}^{cry}	1.0418	0.5209	2.0836
v_{max}^{rev}	0.065746	0.0329	0.1315
v_{max}^{bmal}	0.29055	0.1453	0.5811
kdiss_pc	0.23509	0.1175	0.4702
kdiss_cb	2.2191e-4	1.110e-4	4.438e-4
kass_pc	0.15187	0.0759	0.3037
kass_cb	0.0057803	0.0029	0.0116
Ki_bmal_rev	2.375e-4	1.188e-4	4.750e-5
Ki_cry_rev	0.64066	0.3203	1.2813

2.2 Results of the Sobol' sensitivity analysis

We calculated Sobol' indices in order to assess the relative influence of each parameter. These values measure the relative sensitivity of the outcome to each parameter (first-order) and to all the interactions with the other parameters (total-order). Fig A shows that total output uncertainty is primarily induced by the parameters *kass_pc*, PER-CRY association rate, and *vmax_cry*, *Cry* mRNA maximal transcription rate, and how they interact with the other parameters.

The high sensitivity of D, IL-6, TNF-α and IL-10 to *kass_pc* (Sobol index > 0.5), is due to the consequential role of the complex PER-CRY in the circadian circuitry. Inhibitor proteins PER and CRY dimerize to inhibit their own transcription as well as that of REV-ERB and ROR by acting on CLOCK-BMAL1 protein complex [4]. Overall, D, IL-6 and IL-10 are less sensitive with respect to the parameter set tested (see Table M), whereas TNF-α is more sensitive to parameters affecting CRY and its related complex, PER-CRY.

Fig B shows the time course of the total-order Sobol' indices for *vmax_cry*, *kass_pc* and *kass_cb*, the three most influtential parameters as shown in Fig A. D, IL-6 and IL-10 are sensitive *kass_pc* and *vmax_cry* throughout the inflammation, but the sensitivity of TNF-α to these parameters decreases over time. Moreover, the times of lower sensitivity of IL-10 to *kass_pc*, correspond to the times of higher sensitivity of the cytokine to *kass_cb*.

To assess the robustness of the outcomes to uncertainty in the input parameters in Table M, we plotted the computed simulation results showing the 90% quantile region for D, IL-6, TNF-α and IL-10 (see Fig C). Our results show that the inflammation output variables are qualitatively robust to uncertainty in the parameters that are modified by CJL.

2.3 Assessing sensitivities relative to coupling parameters

We extended our parameter input space to include all the clock model parameters and the coupling parameters. A sensitivity analysis of this larger parameter space allows us to assess how perturbations to the clock parameters can affect the output of the acute inflammation model, regardless of CJL. We performed 5000×(2+ number of input parameters) simulations to derive the Sobol' indices.

Our results indicate that D, IL-6, TNF-α and IL-10 are most sensitive to the coupling parameters, as should be expected. Fig D shows the Sobol' indices for the coupling parameters. These parameters directly

link clock proteins to cytokines, and thus inferences about the effect of parameters on inflammation can be extended to inferences about the associated clock protein on inflammation.

TNF-α is more sensitive to $xTNFCRY$ and $xTNFROR$ as CRY and ROR directly inhibit the production of the cytokine. IL-6 and IL-10 are sensitive to $xIL6REV$ and $xIL10REV$, respectively. This is naturally explained by the direct inhibition of IL-6 and IL-10 by REV-ERB. We note that the damage marker, D, is sensitive to the same parameters as IL-6. This is to be expected because D is upregulated by IL-6.

Fig E shows the time course of the total-order Sobol' indices for the coupling parameters.

IL-6 and Damage. Both outcomes are sensitive to $xIL6REV$ and $xIL10REV$ throughout the duration of inflammation, but are also sensitive to $xTNFCRY$ and $xTNFROR$ at the beginning of inflammation. This indicates that most of the effect of the clock on the damage marker is induced by REV-ERB, but there exists an initial joint effect of REV-ERB, CRY and ROR on IL-6 and D. CRY and ROR act indirectly through their modulation of TNF-α.

TNF-α. The cytokine is particularly sensitive to $xTNFCRY$ and $xTNFROR$. The sensitivity of TNF-α to $xTNFCRY$ decreases as its sensitivity to $xTNFROR$ increases during inflammation. Overall, ROR has a stronger influence on TNF-α compared to CRY. We note also the increased sensitivity of TNF-α to $xIL10REV$ a few hours after the onset of inflammation. This is due to the inhibitory action of IL-10 on TNF-α.

IL-10. Throughout the period of inflammation, IL-10 is most sensitive to $xIL6REV$ and $xIL10REV$. The Sobol' index for $xIL10REV$ decreases shortly after the onset of inflammation, before rising again five hours post-infection. This suggests a role for REV-ERB in the formation of the first peak in IL-10 as well as the second peak that occurs 5h after the beginning of inflammation (see Fig 5 in the manuscript). IL-10 is also sensitive to $xTNFCRY$ and $xTNFROR$ at the start of inflammation. Since ROR and CRY inhibit TNF-α, the sensitivity of IL-10 to those parameters is because TNF-α peaks early during inflammation and activates its production.

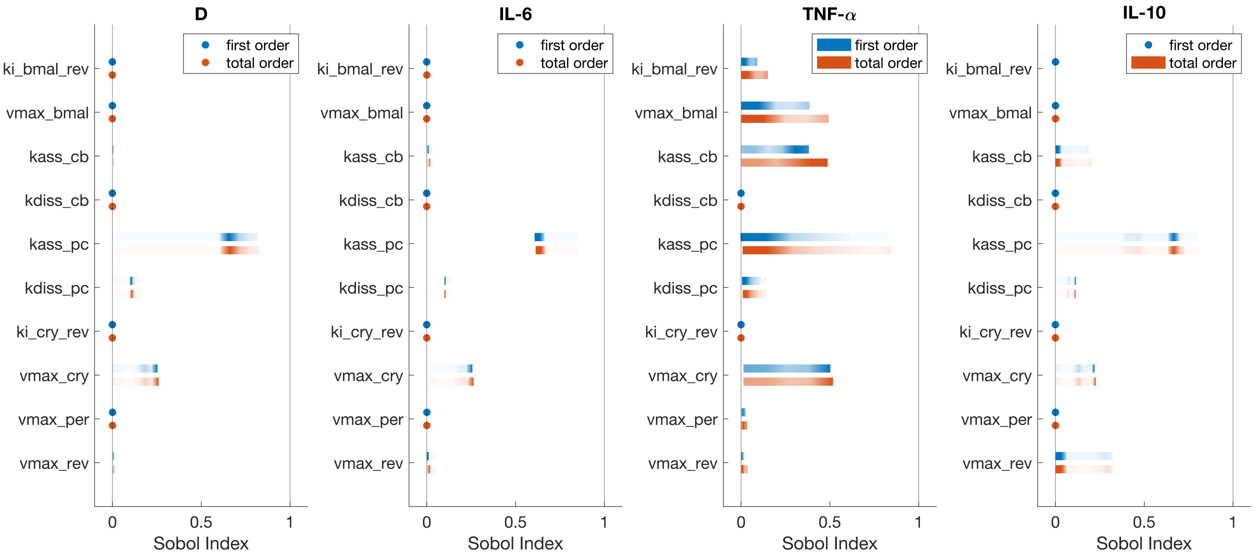

Fig A. Sobol' indices for parameters modified by CJL. Simulation of the baseline coupled model under acute inflammation with endotoxin dose $3mg/kg$. Circles imply no sensitivity to a parameter. A darker area on an index bar indicates sensitivity levels that persisted for most of the simulation time, while faded areas represent sensitivity levels that lasted for shorter periods of time. Infection occurs at CT12.

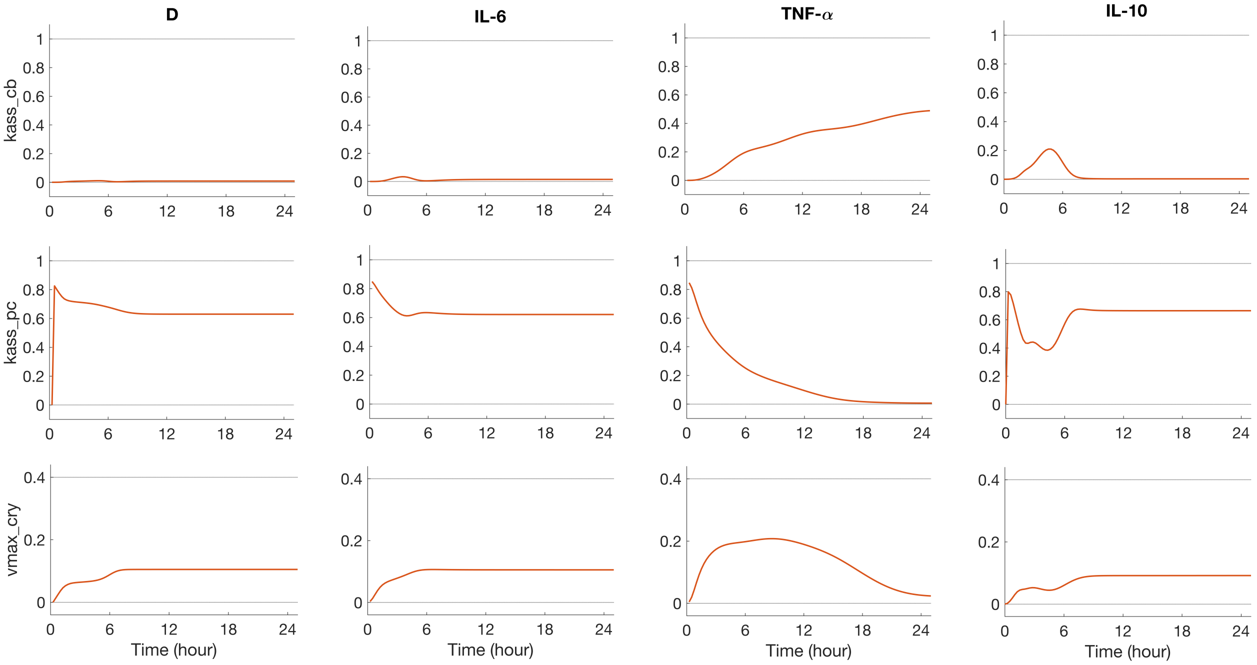

Fig B. Time course of total-order Sobol' indices for vmax_cry, kass_pc and kass_cb. Simulation of the baseline coupled model under acute inflammation with endotoxin dose $3mg/kg$. Infection occurs at CT12.

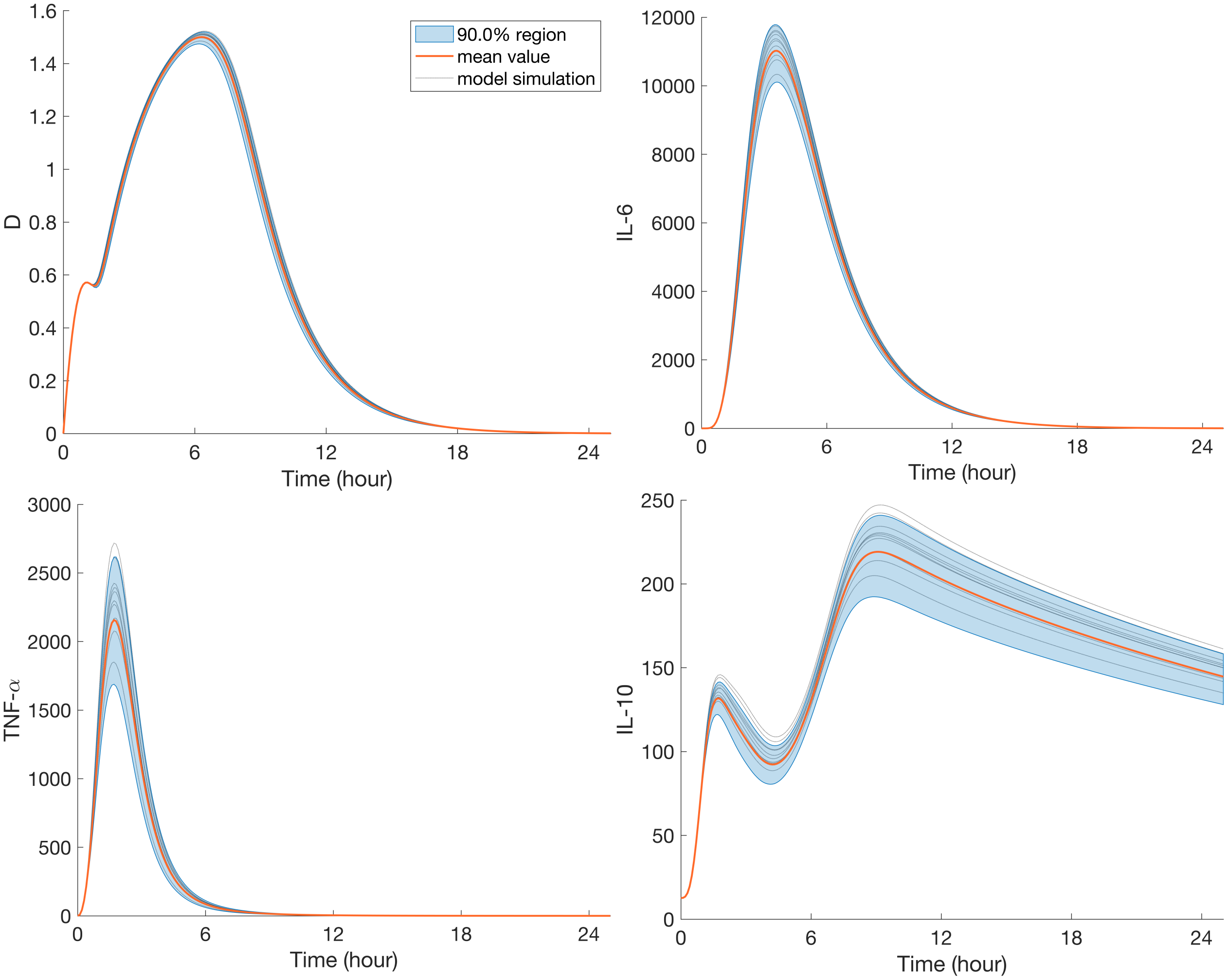

Fig C. Computed simulation results for *D*, *IL-6*, *TNF*-α and *IL-10*. Simulation of the baseline coupled model under acute inflammation with endotoxin dose $3mg/kg$. Infection occurs at CT12. Parameters used in the sensitivity analysis are shown in Table M.

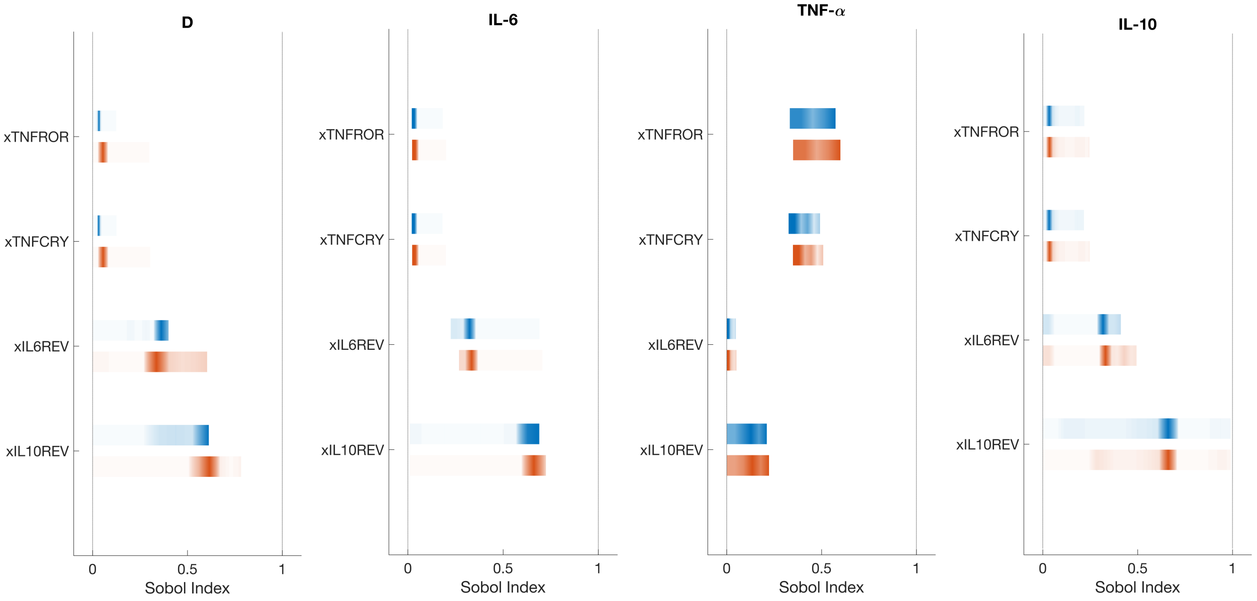

Fig D. Sobol' indices for the coupling parameters. Simulation of the baseline coupled model under acute inflammation with endotoxin dose $3mg/kg$. Circles imply no sensitivity to a parameter. A darker area on an index bar indicates sensitivity levels that persisted for most of the simulation time, while faded areas represent sensitivity levels that lasted for shorter periods of time. Infection occurs at CT12.

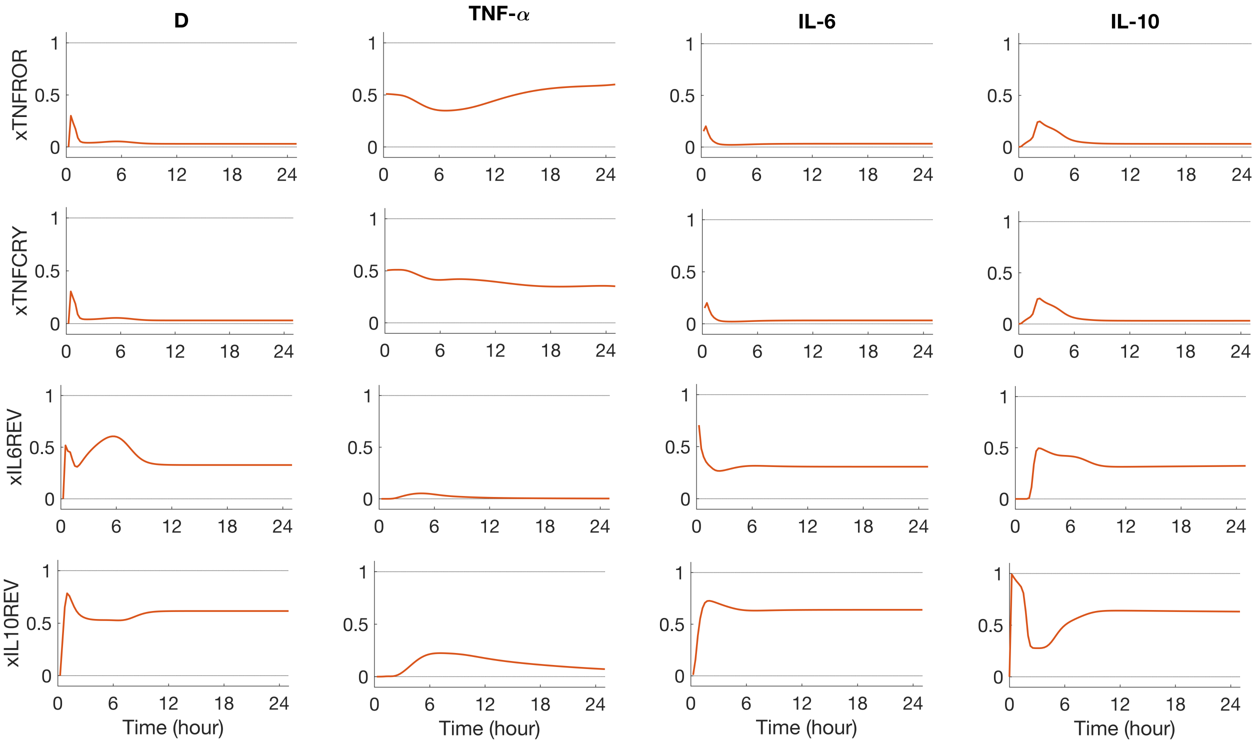

Fig E. Time course of total-order Sobol' indices for the coupling parameters. Simulation of the baseline coupled model under acute inflammation with endotoxin dose $3mg/kg$. Infection occurs at CT12.

References

1. Hadden H, Soldin SJ, Massaro D. Circadian disruption alters mouse lung clock gene expression and lung mechanics. J Appl Physiol. 2012;113(3):385–392. doi:10.1152/japplphysiol.00244.2012.

2. Sobol' IM. On sensitivity estimation for nonlinear mathematical models. Matematicheskoe modelirovanie. 1990;2(1):112–118.

3. Nossent J, Elsen P, Bauwens W. Sobol'sensitivity analysis of a complex environmental model. Environmental Modelling & Software. 2011;26(12):1515–1525.

4. Dibner C, Schibler U, Albrecht U. The mammalian circadian timing system: organization and coordination of central and peripheral clocks. Annu Rev Physiol. 2010;72:517–549.
